# Redox-independent chromium isotope fractionation induced by ligand-promoted dissolution

**DOI:** 10.1038/s41467-017-01694-y

**Published:** 2017-11-17

**Authors:** Emily M. Saad, Xiangli Wang, Noah J. Planavsky, Christopher T. Reinhard, Yuanzhi Tang

**Affiliations:** 1 School of Earth and Atmospheric Sciences, Georgia Institute of Technology, Atlanta, GA 30332 USA; 2 Department of Geology and Geophysics, Yale University, New Haven, CT 06511 USA

## Abstract

The chromium (Cr) isotope system has emerged as a potential proxy for tracing the Earth’s atmospheric evolution based on a redox-dependent framework for Cr mobilization and isotope fractionation. Although studies have demonstrated that redox-independent pathways can also mobilize Cr, no quantitative constraints exist on the associated isotope fractionations. Here we survey the effects of common environmental ligands on the dissolution of Cr(III)-(oxy)hydroxide solids and associated Cr isotope fractionation. For a variety of organic acids and siderophores, δ^53^Cr values of dissolved Cr(III) are −0.27 to 1.23‰, within the range of previously observed Cr isotope signatures in rock records linked to Cr redox cycling. Thus, ligand-promoted dissolution of Cr-containing solids, a redox-independent process, must be taken into account when using sedimentary Cr isotope signatures to diagnose atmospheric oxygen levels. This work provides a step towards establishing a more robust framework for using Cr isotopes to track the evolution of the Earth’s atmosphere.

## Abstract (web summary)

The chromium (Cr) isotope system has emerged as a potential proxy for tracing Earth’s atmospheric evolution based on a redox-dependent framework. Here the authors show that ligand-complexation, a redox-independent process, must be considered when using Cr isotope signatures to diagnose atmospheric oxygen levels.

## Introduction

The chromium (Cr) isotope system has been exploited as a highly discriminating tracer of redox processes in the Earth’s surface environments, and is under development as a novel paleobarometer for atmospheric oxygen levels^1–5^. Many of these applications are based on the assumption that Cr isotope fractionations are linked predominantly to Cr redox transformations. Consistent with this view, theoretical and experimental studies have suggested very little Cr fractionation during redox-independent processes, such as adsorption^6^, though recent experimental studies demonstrated large equilibrium fractionations between various Cr–Cl species^7^. Cr isotopes have been extensively employed in environmental geochemistry as a quantitative proxy for Cr transport and remediation efficiency (see ref. ^8^ and references therein). However, in order to quantitatively apply this isotope system as a tracer for Earth’s surface processes, it is critical to more precisely quantify a wider range of factors controlling the global Cr cycle and isotope budget^9^. Indeed, since the early proposed uses of the Cr isotope system, there have been several studies recognizing the need to account for other biogeochemical processes in addition to the redox pathway-based framework of this isotope system^7,10–14^.

The two most common oxidation states of Cr in natural environments are Cr(III) and Cr(VI). Cr(III) is typically insoluble and is the most common form of Cr in rocks and minerals at the Earth’s surface. Cr(VI) is typically soluble and is the dominant form of mobile Cr in natural environments. The standard view of the Cr isotope paleoredox proxy is grounded in the idea that oxidative weathering of Cr(III)-containing minerals by manganese (Mn) oxides, the formation of which requires free oxygen, is required for Cr redox cycling and Cr isotope fractionations. During this oxidation process, soluble Cr(VI) is released and transported to oceans, reduced to Cr(III) under reducing environments, and deposited in marine sediments^1,15^. Thus, authigenic marine sediments capture an integrated isotopic signature reflective of the redox processes involved in Cr(III) oxidative mobilization, downstream Cr(VI) reduction, and ultimate Cr(III) burial in sediments^1^.

The redox transformation between Cr(III) and Cr(VI) causes a significant change in the local coordination environment of Cr. Cr(III) typically occurs as an octahedrally coordinated cation, whereas Cr(VI) primarily occurs as a tetrahedrally coordinated oxyanion. The large change in coordination environment during redox transformations leads to significant isotopic fractionation, similar to other redox-sensitive elements^16^. Unweathered igneous rocks, with limited exceptions^17^, exhibit a narrow range of δ^53^Cr values of −0.124 ± 0.101‰ (defined as δ^53^Cr = [(^53^Cr/^52^Cr)_sample_/(^53^Cr/^52^Cr)_standard_ −1] × 1000)^15^. Cr(III) oxidation to Cr(VI) can result in significant isotopic fractionations, both negative and positive (^53^ε_Cr(VI)-Cr(III)_ ranging from −2.5 to +1.1‰), depending on the reaction conditions; while reduction of Cr(VI) to Cr(III) has consistently been shown to leave the remaining Cr(VI) isotopically heavy (^53^ε_Cr(III)–Cr(VI)_ ranging from −7.6 to −0.4‰) (see, e.g., ref. ^8^). Because the isotopic exchange and subsequent isotopic equilibrium between Cr(III) and Cr(VI) requires three electron transfer with a significant coordination reconfiguration, the isotopic exchange rate of these species is extremely slow^18,19^. However, when dissolved Cr(VI)- and Cr(III)-containing solids are in contact for long periods, equilibrium fractionation may become relevant. The significance of such a process under actual environmental settings (e.g., during the weathering of Cr-containing rocks/minerals) can vary and is dependent on many factors, such as the relative ratio of the exchanging Cr(VI) and Cr(III) species^19^. Therefore, Cr isotope systematics during the Earth’s surface processes are generally assumed to be dominated by kinetic fractionations. Regardless, current experimental and empirical results suggest that both kinetic and equilibrium processes tend to lead to Cr(VI) that is isotopically heavy^19,20^.

One generally overlooked mechanism for Cr(III) mobilization is ligand complexation. Indeed, measurements on natural waters have revealed elevated concentrations of dissolved Cr(III) that are higher than the solubility of Cr(OH)_3_ solids, one common sink phase of Cr(III), and suggest the likely complexation of Cr(III) by inorganic^21^ and/or organic molecules (e.g., refs. ^22–24^). The speciation of Cr in hydrothermal fluids, and marine settings more broadly, is also thought to be influenced by organically complexed Cr(III)^25,26^. Two classes of organic ligands, siderophores (e.g., desferrioxamine B, rhizoferrin, protochelin)^27^ and organic acids (e.g., citric acid, ethylenediaminetetraacetic acid, nitrilotriacetic acid)^28^, have been shown to be capable of solubilizing Cr(III) from the Cr(OH)_3_ solid phase under environmentally relevant conditions. Both siderophores and organic acids are ubiquitous organic molecules in natural environments and are produced by a wide range of microorganisms and often co-exuded^29^. Siderophores are organic chelating agents with a high affinity for Fe(III) for enhancing Fe dissolution from low-solubility Fe(III)-mineral phases^30^. Siderophores have also been shown to have strong affinities for other trivalent metal cations such as Cr(III) due to their structural similarity (e.g., size, coordination environment) to Fe(III)^31^. Organic acids are known to contribute to the mobilization of metals in soils^32^, and a wide range of organic acids (e.g., oxalic, citric, fulvic, and humic acids) have been shown to complex with Cr(III)^33–35^. Organic acids are likely to have been present throughout the Earth’s history through abiotic or biotic synthesis pathways (see, e.g., refs. ^36, 37^) and may have played important roles in the weathering^38^ of poorly soluble transition metals.

Despite the potential importance of Cr(III)–ligand complexes in Earth’s surface, Cr isotope fractionation during Cr(III) complexation with organic ligands has not been experimentally measured, although the potential for fractionation upon complexation with inorganic ligands has been previously suggested by theoretical studies^19,20^ and observed experimentally^7^. Additionally, several recent studies have inferred redox-independent Cr cycling in the interpretation of relevant rock records based on the site-specific geochemistry and Cr isotope dynamics^10,11^.

Here, we survey the extent of Cr isotope fractionation that occurs during ligand complexation and dissolution of Cr(III)-containing solid phases. Given the potential for analytically significant fractionations, these redox-independent processes may add complexity when using Cr isotopes to track Cr oxidative mobilization and Cr reduction during environmental remediation. We systematically characterize the dissolution of a Cr(III)-containing solid by a wide range of organic ligands, and quantified the attendant isotope effects with an eye toward better understanding the potential impact of Cr(III)–ligand complexation on global Cr cycling and interpretation of isotope signatures.

## Results

### Ligand promoted dissolution

**Fig. 1:**
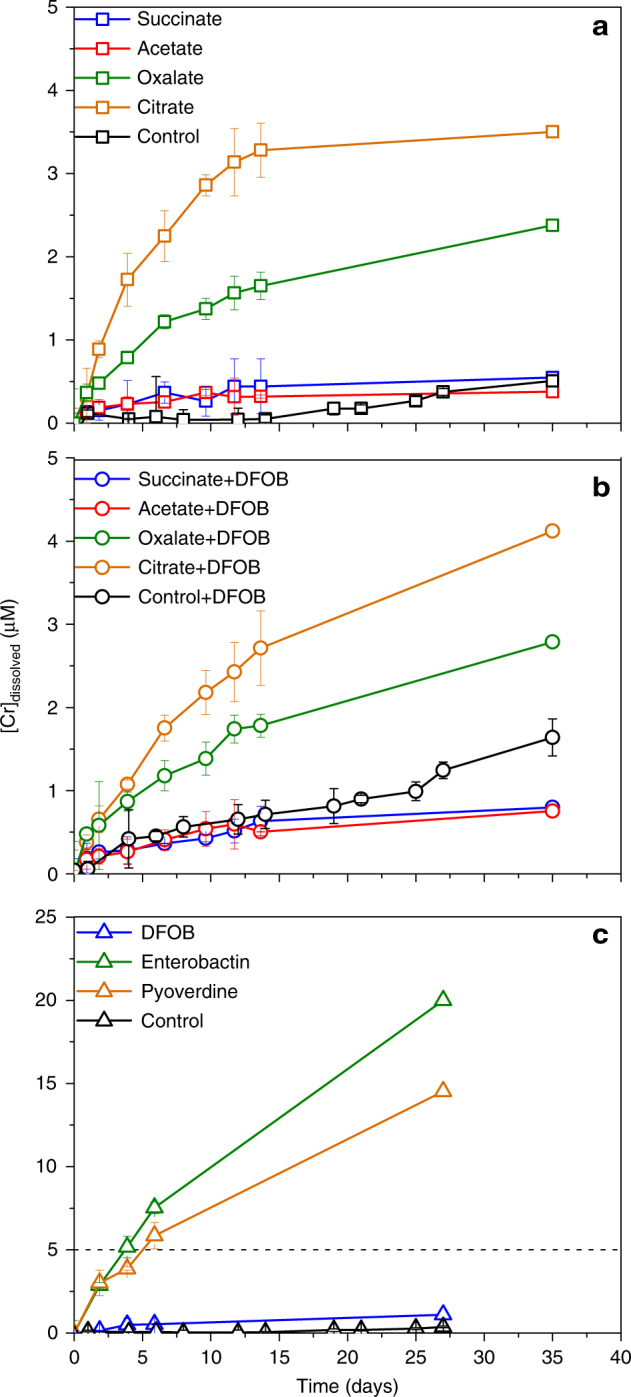
Cr dissolution profile in the presence of different ligands. Dissolved Cr concentrations in the presence of **a** organic acids only, **b** organic acids and the siderophore, desferrioxamine B (DFOB), and **c** siderophores only. Experimental conditions are 0.2 g l^−1^ of Cr(OH)_3_ at pH 7 (10 mM HEPES), and ionic strength of 0.1 M. Error bars represent the standard deviation between replicates. The dashed line in (**c**) indicates the maximum value of the *y*-axis in (**a**) and (**b**). All dissolved Cr was determined to be in the +3 oxidation state

In the absence of organic ligands, dissolved Cr remained <0.5 µM throughout the experiment with detectable release only occurring after 14 days (Fig. 1a). The presence of organic ligands promoted the dissolution of Cr to variable extents (Fig. 1). In the presence of organic acids alone, citrate released the greatest amount of Cr, reaching a plateau at 3.5 μM, followed by oxalate, which reached 2.4 μM by the end of the experiment (Fig. 1a). The presence of a dissolution plateau is possibly due to surface passivation. Succinate and acetate promoted Cr release to a similar extent as the control (i.e., no organic ligands), reaching 0.5 and 0.4 μM, respectively, at the end of experiments, both with detectable Cr throughout the experiment (whereas in control experiments dissolved Cr was only detected after ~ 15 days). The presence of both desferrioxamine B (DFOB) and organic acids resulted in similar dissolution trends as the organic acid-only treatments (Fig. 1b). Citrate, followed by oxalate, induced the greatest amount of dissolved Cr, reaching 4.1 and 2.7 μM, respectively, and with continuous increase at the end of the experiment. The addition of succinate and acetate with DFOB both resulted in an ~ 0.8 μM dissolved Cr at the end of the experiment, less than in the presence of DFOB alone (1.5 μM). Cr dissolution was also dependent on siderophore type (Fig. 1c). Enterobactin resulted in the greatest amount of dissolved Cr (20 μM), followed by pyoverdine (15 μM) and DFOB (1.5 μM). None of the siderophore-only treatments reached a concentration plateau by the end of the experiment.

**Fig. 2:**
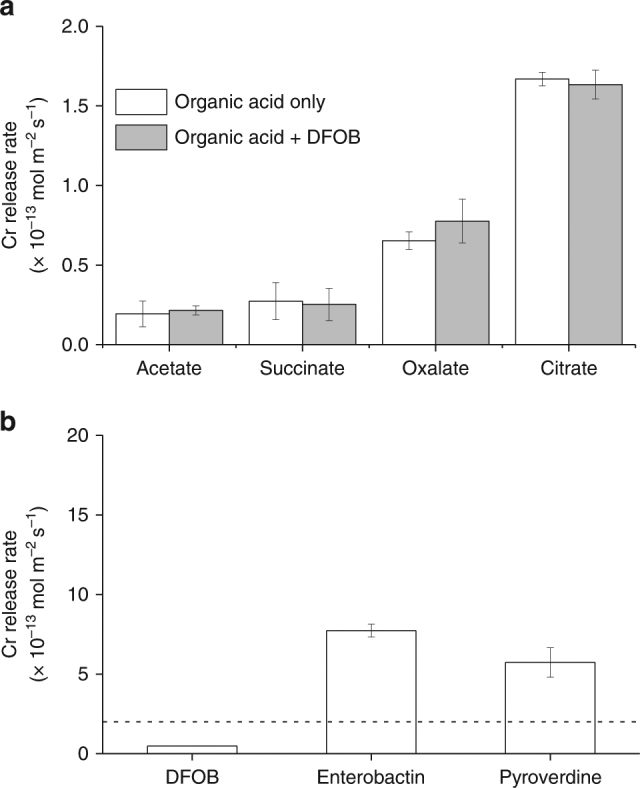
Cr dissolution rate in the presence of different ligands. The initial surface area normalized release rates of Cr from Cr(OH)_3_ in the presence of **a** organic acids with and without the siderophore, desferrioxamine B (DFOB), or **b** siderophores only. The dashed line in (**b**) is at the maximum *y*-value of (**a**). The average rates of replicates are presented with error bars reflecting standard deviation between replicates. Experimental conditions are 0.2 g l^−1^ of Cr(OH)_3_ at pH 7 (10 mM HEPES) and ionic strength of 0.1 M

Both siderophores and organic acids increased the initial Cr release rates (surface area normalized) relative to the absence of a ligand, for which an initial dissolution rate could not be calculated by linear regression (*R* ^2^ = 0.2) (Fig. 2 and Supplementary Table 1). In the presence of organic acids, initial Cr release rates were highest for citrate (1.6 ± 0.1 × 10^−13^ mol m^−2^ s^−1^), followed by oxalate (6.5 ± 0.6 × 10^−14^ mol m^−2^ s^−1^), and succinate and acetate (both ~ 2 × 10^−14^ mol m^−^ s^−1^) (Fig. 2a and Supplementary Table 1). The combined presence of DFOB and organic acids did not significantly affect the dissolution rate relative to experiments with organic acids alone. In the presence of siderophores alone, initial Cr release rates were highest for enterobactin (7.7 ± 0.4 × 10^−13^ mol m^−2^ s^−1^), followed by pyoverdine (5.7 ± 0.9 × 10^−13^ mol m^−2^ s^−1^), and finally DFOB (4.8 ± 0.1 × 10^−14^ mol m^−2^ s^−1^) (Fig. 2b and Supplementary Table 1). In general, organic acids released less Cr into solution than siderophores. The exception is citrate, which resulted in a dissolution rate around 3 times greater than that of DFOB.

### Cr isotopic composition

**Fig. 3:**
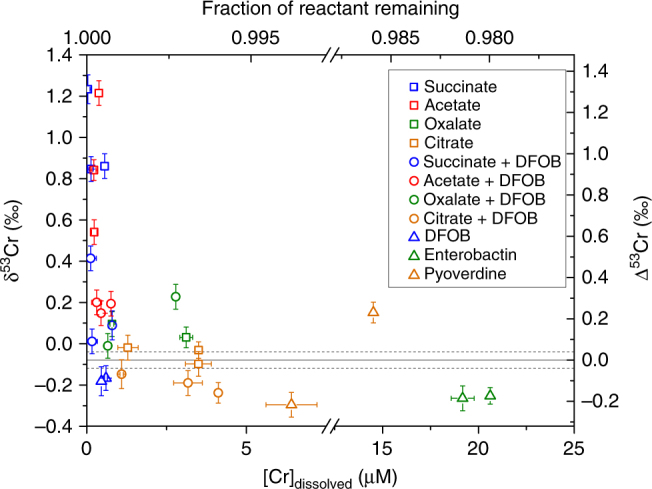
Cr isotope composition. δ^53^Cr as a function of reaction extent (either fraction of reactant remaining or concentration of Cr dissolved) for each ligand or ligand combination. Δ^53^Cr represents δ^53^Cr_solution_−δ^53^Cr_initial solid_ at distinct points during dissolution. The isotopic signature of the starting solid is represented by the solid line at δ^53^Cr = −0.08‰ and Δ^53^Cr = 0. The dashed lines represent ±2σ (analytical measurement reproducibility). Experimental conditions are 0.2 g l^−1^ of Cr(OH)_3_ at pH 7 (10 mM HEPES) and ionic strength of 0.1 M

The Cr isotopic composition of the dissolved Cr(III) was also examined throughout the experiments (Fig. 3). A wide range of δ^53^Cr values were observed in the solubilized Cr, from +1.23 to −0.27‰. The δ^53^Cr values varied based on ligand type and reaction extent. The isotope signature of dissolved Cr approaches that of the original solid with increasing reaction extent for the experiments with organic acids only. Interestingly, though the influence of DFOB on organic acid-promoted dissolution was not obvious with regard to Cr(III) release rates, a significant impact on the magnitude of isotopic fractionation was observed when comparing DFOB+organic acid experiments to organic acid-only experiments.

## Discussion

Organic ligands are known to promote the dissolution of metal oxides by forming inner sphere metal–ligand complexes at the solid–solution interface and weakening the metal–oxygen bond on the solid surface. The structure of the ligand influences the stability of both the surface and dissolved metal–ligand complexes and consequently the rate of dissolution. The rate-limiting step during ligand-promoted dissolution is the detachment of the metal–ligand complex from the surface; thus, increasing stability of the dissolved metal–ligand complex leads to increasing dissolution rate^39^. The stability constants of Cr(III)-siderophore complexes are not well established due to the slow ligand exchange rate, weak ultraviolet–visible (UV–vis) absorbances, and low reduction potentials, all of which prevent traditional methods for the determination of stability constants (see ref. ^27^ and references therein). Because limited stability data are available for Cr(III)–ligand complexes for the ligands studied here, the following discussions will use the stability constants of Fe(III)–ligand complexes to estimate the potential influences of such factor on dissolution and isotope fractionation. The rationale for using Fe(III)–ligand complex stability constants as proxies is based on the similar strength of interaction between Fe(III) or Cr(III) cations and a ligand containing a hard Lewis base (i.e., the hardness of the cation is similar)^40^. Therefore, the ionic potential of metal cations (i.e., valence divided by ionic radius) positively correlates with the metal–ligand stability constant^27,40^. The similarity of the ionic potential of Fe(III) (ionic radius of 0.645 Å) to Cr(III) (ionic radius of 0.615 Å)^41^ allows for an appropriate first estimation of Cr(III)–ligand trends. Although a more complete estimation of the Cr(III)–ligand stability constants would require the metal–ligand stability constants for a greater number of metals^27,40^, we suggest using Fe(III) stability constants as a first-order approximation of the corresponding Cr(III)–ligand complex stabilities. However, it should be noted that in some instances the measured values of Cr(III)–ligand stability, specifically for siderophores, do not necessarily follow the correlating Fe(III)–ligand stability trends, as has been observed for rhizoferrin, an α-hydroxycarboxylate-type siderophore, where the Cr(III)–rhizoferrin stability constant is 4 orders of magnitude higher than for the corresponding Fe(III)–rhizoferrin complex^27^. The difference in siderophore affinity has also been previously noted between Fe(III) and Mn(III) due to the identity of the binding moiety of the siderophore and the electron configuration of the metal atoms^42^.

**Fig. 4:**
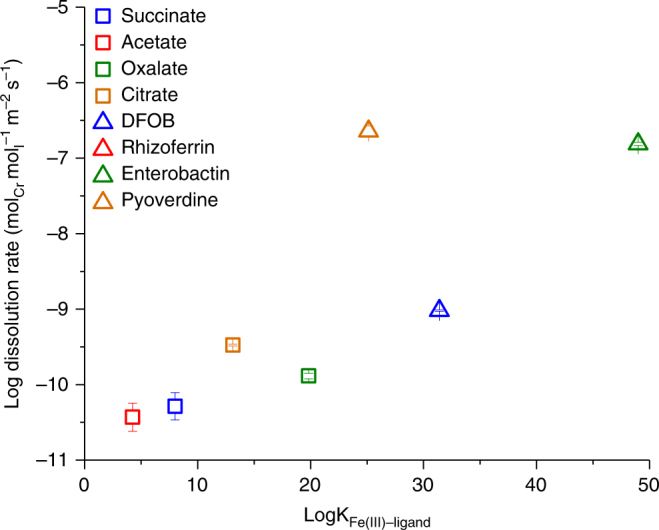
Cr dissolution rate vs metal–ligand stability constants. Initial dissolution rates of 0.2 g l^−1^ of Cr(OH)_3_ (mol Cr mol ligand^−1^  m^−2^ s^−1^) at pH 7 and ionic strength of 0.1 M as a function of the stability constant of the corresponding Fe(III)–ligand complexes. Stability constants are for Fe(III)–organic acid complexes^44^ predicted to be the most thermodynamically stable under these conditions using MINTEQ^43^. Fe(III)–siderophore stability constants are for the Fe(III)–siderophore complex with a completely unprotonated ligand (see ref. ^45^ and references therein). Fe(III)–ligand stability constants are presented for consistency due to the similarity in ionic potential between Fe(III) and Cr(III), because Cr(III)–ligand stability constants are not available for all ligands

In the presence of organic acids, we find that dissolution rates generally increase with increasing stability constant of the corresponding Fe(III)–ligand complex (Fig. 4 and Supplementary Fig. 1)^43–45^. Of the organic acids utilized here, citrate, a strong chelating ligand, exhibited the greatest dissolution rate at 0.27 ± 0.01 μM d^−1^ (1.67 ± 0.04 × 10^−13^ mol m^−2^ s^−1^; change in units in order to offer a consistent comparison), consistent with the previously reported rate of citrate-promoted Cr(OH)_3_ dissolution under similar experimental conditions (0.22 ± 0.09 μM d^−1^)^28^. Although the rates of organic acid-promoted dissolution of Cr(OH)_3_ for oxalate, succinate, and acetate have not been reported to our knowledge, inference can be made based on the trends observed with Fe oxides. For example, similar trends of dissolution rates have been observed for organic acid-promoted ferrihydrite dissolution at pH 7, which decreased in the order of citrate, oxalate, and succinate^46^. These trends can be described by the binding mechanism exhibited by each ligand. Citrate has been shown to adsorb to Fe oxides with each carboxyl group bound to different Fe(III) sites on the solid surface, leading to its effectiveness at dissolving metal oxides^47^. Oxalate has been shown to form a bidentate mononuclear complex with Fe(III) in hematite, while succinate formed a (weaker) monodentate surface complex, leading to the observed differences in dissolution rates^48^. Acetate is a monodentate ligand that has been shown to preferentially occupy adsorption sites on metal oxide surfaces^28^, though is not expected to significantly affect the dissolution rate^49^. In general, the trends in organic acid-promoted dissolution observed in this study are consistent with expectations.

The dissolution of Cr(OH)_3_ by siderophores has recently been studied at several pH values by Duckworth et al.^27^. The average surface area normalized dissolution rates at pH 7 and under similar experimental conditions (0.2 g l^−1^ Cr(OH)_3_, 100 μM siderophore, 10 mM 4-(2-hydroxyethyl)-1-piperazineethanesulfonic acid (HEPES)) were 4.3 ± 0.7 × 10^−13^ mol m^−2^ s^−1^ for DFOB and 3.0 ± 1.7 × 10^−12^ mol m^−2^ s^−1^ for protochelin (a triscatecholate-type siderophore like enterobactin)^27^, both of which are within a factor of 10 of rates observed in this experiment (Fig. 2 and Supplementary Table 1). The differences in observed rates may be the result of differences in properties of the initial synthesized Cr(OH)_3_ solids (i.e., aggregation) and the difference in the number of time points considered. Unlike organic acids, the relationship between stability constant and dissolution rate of the siderophores does not appear to be correlated (Fig. 4 and Supplementary Fig. 1)^43–45^, similar to what was observed in Duckworth et al.^27^. However, as mentioned previously, siderophore observations with Cr may not be explicitly comparable to the Fe(III)–siderophore stability constants. For the ligands with an available Cr-ligand stability constant and dominant species at the experimental conditions, Cr-acetate (logK = 15)^43,44^ and Cr-DFOB (logK = 33)^27^, general agreement between the Cr-ligand and Fe-ligand stability constant correlation with stability constant is observed. While Fe oxide dissolution rates have been shown to positively correlate to the Fe(III)-ligand stability constant^50^, Cr(III)–siderophore complexes have been shown to behave differently from their corresponding Fe(III)–siderophore complexes^27^. It is important to note that we focus here on dissolution rates rather than dissolution rate constants, which may result in some discrepancies due to siderophore adsorption affinities^51^, which were not the focus of this study.

**Fig. 5:**
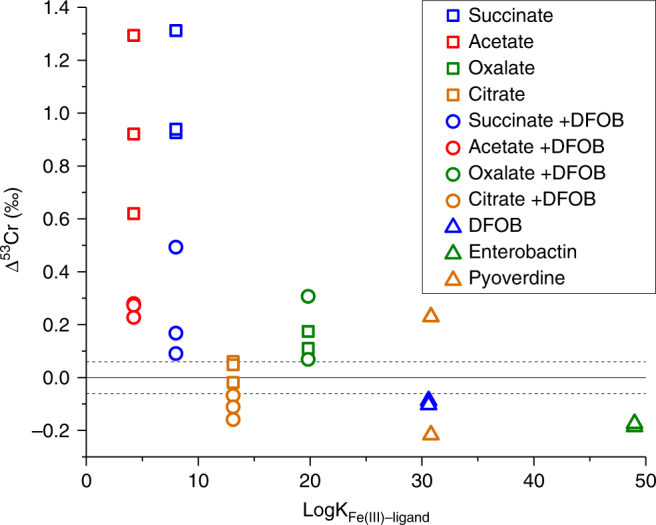
Cr isotope fractionation vs metal–ligand stability constant. Δ^53^Cr (defined as Δ^53^Cr = δ^53^Cr_solution_−δ^53^Cr_starting solid_) at pH 7 and ionic strength of 0.1 M as a function of the stability constant of the corresponding Fe(III)–ligand complex. The data points for each condition represent complete harvests at various times throughout the experiment. Stability constants are for Fe(III)–organic acid complexes^44^ predicted to be the most thermodynamically stable under these conditions using MINTEQ^43^. Fe(III)–siderophore stability constants are for the Fe(III)–siderophore complex with a completely unprotonated ligand (see ref. ^45^ and references therein). This plot is presented with the caveat that not all dissolved metal may be ligand complexed. Fe(III)–ligand stability constants are presented for consistency as Cr(III)–ligand stability constants are not available for all ligands and due to the similarity in ionic potential between Fe(III) and Cr(III). No observed isotopic fractionation is indicated by the solid line at Δ^53^Cr = 0. The dashed lines represent ±2σ

The stability constants of the metal–ligand complexes can also be correlated with the observed isotopic fractionation factors (Fig. 5)^43–45^. Metal–ligand complexes with the largest stability constants (based on stability constants for the corresponding Fe(III)–ligand) resulted in less positive or more negative fractionation (Fig. 5)^43–45^. For the ligands with an available Cr-ligand stability constant and dominant species at the experimental conditions, Cr-acetate (logK = 15)^43,44^ and Cr-DFOB (logK = 33)^27^, Cr-acetate does not follow the predicted trend while Cr-DFOB does. A similar trend has been observed with Fe isotopes during organic acid-promoted dissolution of hornblende^52^. However, in that case the trend encompassed greater fractionation with increasing stability constant, opposite of the observations of this study. If the predominant control is kinetic isotope fractionation, we would expect lighter isotopes (i.e., negative Δ^53^Cr defined as Δ^53^Cr = δ^53^Cr_solution_−δ^53^Cr_starting solid_) to enrich in solution at low dissolved Cr concentrations and approach the isotopic signature of the starting solid during continued dissolution^16^. In this study, maximum isotopic fractionation was observed relatively soon after the experiment was initiated with isotope values eventually approaching the bulk solid isotope signature at greater dissolved Cr, similar to what has been previously observed with Fe isotopes during dissolution experiments on Fe oxides^53^. However, the enrichment of lighter isotopes in the solution during the early stages of dissolution was not evident in our system; instead, positive values for Δ^53^Cr were observed during initial dissolution (Fig. 3). If the Cr(III)–oxygen bond(s) in the ligand is stronger than the Cr(III)–oxygen bonds in the solid, one would expect to see the observed enrichment of heavier isotopes (i.e., positive Δ^53^Cr) in the dissolved fraction (Fig. 3). However, Δ^53^Cr does not positively correlate to the predicted stability constant of the Fe(III)–ligand complexes, but instead generally exhibits a negative correlation (Fig. 5)^43–45^. The highly positive Δ^53^Cr values in the treatments with low stability constant ligands (e.g., acetate) may be caused by a fractionation during back reaction of weakly complexed liberated Cr(III). This process could be insignificant in more strongly bound Cr(III). Furthermore, if dissolution occurs layer by layer such that ligands complex and dissolve surface Cr atoms completely before the next layer is exposed and solubilized, a large fractionation may only be observed in the early stages of dissolution^16^. Given that Cr(OH)_3_ only exhibits a particle size of < 10 Å and is likely a polymer of only a few CrO_6_ octahedra^54^, rind effects are expected to be minimal for individual particles. However, since heavy aggregation is typically observed for these nanoparticles, rind effects might be more significant at an aggregate level.

Overall, results from this study have important implications for research using the Cr isotope system as a paleoredox and environmental tracer. Dissolution experiments were conducted at pH 7 in order to isolate the effects of ligand-promoted dissolution on Cr(OH)_3_ solids. Therefore, despite the lower rates and extents of dissolution relative to proton-promoted dissolution^27^, ligand-promoted dissolution of Cr-containing phases might contribute significantly to Cr mobilization under nonacidic conditions, such as in the marine environment. For instance, isotopically fractionated Cr(III) may be leached from dusts or sourced from a benthic flux of organically complexed Cr(III). Again, the presence of ligand-bound Cr(III) in the modern oceans supports ligand complexation as an important component of the Cr cycle^22,25,26^. Terrestrial waters can also be near neutral or alkaline, suggesting the need to survey for significant ligand complexed Cr(III) when using Cr isotopes to monitor Cr reduction in environmental remediation studies.

**Fig. 6:**
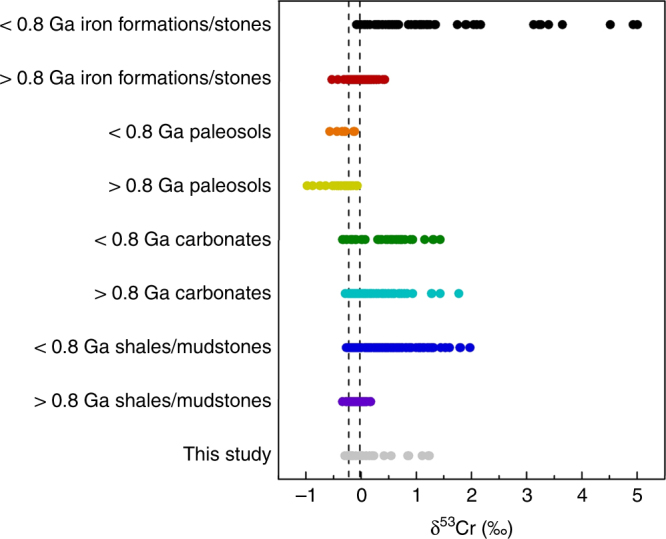
Cr signatures from this study compared to field data. Redox-independent Cr isotope signatures observed in this study compared with previously observed δ^53^Cr ranges from various archives: iron formations/stones^1,3,4,56^, paleosols^3,57^, carbonates^14,56^, and shales/mudstones^2,4,5,9,55^. The dashed lines delineate the δ^53^Cr ranges of igneous rocks (mainly basalts and granites)^15^

The redox-independent Cr isotope fractionations observed in this study are within the range of values previously reported to reconstruct oxygenation events (Fig. 6)^1–5,9,14,15,55–57^. Therefore, the results from this study bolster the case that the interpretation of the Cr isotope proxy may need to be revisited in some circumstances to account for redox-independent processes, such as the mobilization of Cr(III) by organic or inorganic complexing ligands. As previously suggested^10,11,14^, some sedimentary Cr isotope archives are more likely to be shaped by ligand-bound Cr and attenuating isotope fractionations associated with organic stabilization. As mentioned above, alkaline systems (e.g., marine systems) may be more likely to express the effects of ligand-promoted solubilization than low pH systems, given that proton promoted dissolution scales with pH. Marine systems removed from a terrestrial source (e.g., carbonate platforms) might be expected to capture variably fractionated organically or inorganically complexed Cr(III), such as dissolved Cr(III)–carbonate complexes which have also shown to be significant during ultramafic rock weathering^21^. However, given the high density of organic acids in the weathering realm (see, e.g., ref. ^38^), soils (and thus paleosols) with evidence for intense weathering may also be susceptible to expression of Cr(III)–organic isotope fractionations (see, e.g., ref. ^10^). Therefore, we suggest care should be taken when interpreting negative δ^53^Cr values in paleosol and ancient carbonate rocks as indication of redox cycling of Cr^3,10,58^.

The common occurrence of organic-bound Cr(III) in modern environments and the potential for the formation of organic acids throughout the Earth’s history indicates that the presented results must be considered when using Cr isotopes to track the Earth’s oxygenation. Further, the presence of both positive and negative Δ^53^Cr values will make it difficult to directly assess the influence of Cr(III)–ligand isotope effects in deep time studies. However, we emphasize that this does not require abandonment of the basic framework for using Cr isotopes as a paleoredox proxy, where large Cr isotope variations in the sedimentary records (e.g., strong deviation from igneous rocks) are a signal for oxidative Cr cycling that can be linked to atmospheric oxygen levels^1,3,5^. The common occurrence of authigenic but isotopically unfractionated Cr in ancient sedimentary rocks^1,4,5^ implies that Cr(III) moving through the Earth’s surface is not ubiquitously isotopically fractionated by ligand-promoted solubilization, though some small variability is observed in each of these studies. In this light, the possibility of some variable Cr isotope values should be expected in low oxygen systems (e.g., without Cr(III) oxidation), and moving forward, the paleoredox Cr isotope framework must account for this possibility. Despite this added complexity, we suggest that organic complexation is unlikely to lead to a systematic shift in the mean δ^53^Cr value of the mobile Cr reservoir on a global scale (i.e., the average δ^53^Cr value of dissolved Cr in freshwater and marine systems), in contrast to extensive Cr oxidation, which can result in large ^53^Cr-enriched surface reservoirs as observed on the modern and Phanerozoic Earth^12,13,59–63^. Nevertheless, our results imply that some Cr isotope data from individual formations may lead to ambiguous conclusions about the presence of oxidative Cr cycling.

**Fig. 7:**
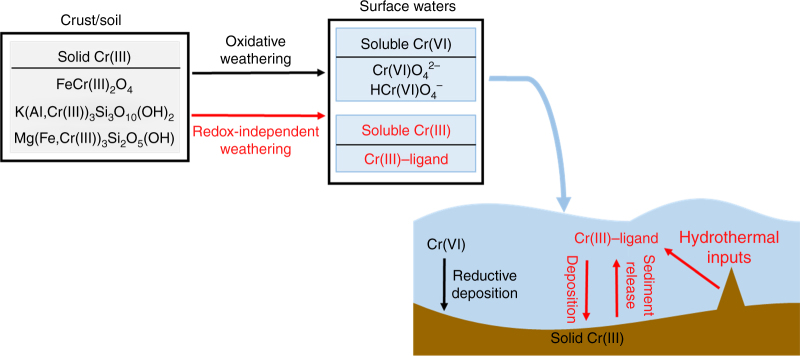
Suggested updated Cr cycle. Schematic of Cr cycle as a paleobarometer for atmospheric oxygen level. Red text and arrows indicate pathways contributing to isotope fractionation that are currently unconstrained, but important in calibration of this system

Our data provide a step towards accounting for Cr isotopic variability in the Earth’s rock record by pointing toward specific non-redox processes that must be considered in the calibration of the Cr isotope system as a paleoredox proxy. Our results strongly suggest that fractionations during both redox-dependent and redox-independent Cr cycling need to be assessed (Fig. 7). In particular, organic ligands can interact with Cr during both continental weathering and after sediment deposition. However, observations from the existing geochemical record and modern natural waters suggest that extensive Cr redox cycling remains the most effect means for generating significant, large-scale Cr isotopic variability at Earth’s surface.

## Methods

### Selection of solid phase and ligands

Amorphous Cr(III)-(oxy)hydroxide (hereafter referred to as Cr(OH)_3_) was selected as a representative compound for solid-phase Cr in natural environments as it is a common Cr(III)-containing solid phase, and an endmember in the Cr(III)-Fe(III)-(oxy)hydroxide solid solution series^54^. A variety of organic ligands and their impacts on Cr(OH)_3_ dissolution and isotope fractionation were surveyed. Siderophores and organic acids were studied as they are two common classes of metal-complexing organic ligands in the natural environment as previously discussed. Siderophores are generally divided into three primary types based on the characteristic metal binding functional group, including α-hydroxycarboxylic acid (hydroxycarboxylate type), catechol (catecholate type), and hydroxamic acid (hydroxamate type)^64^. The most common bacterial and fungal siderophores are catecholates, and hydroxamates, respectively, though other types, including a mixed type, are also quite common and exist with a variety of chemical structures^64^. We chose the following types of siderophores to cover a range of binding types: DFOB (hydroxamate type), enterobactin (catecholate type), and pyoverdine (mixed type) (Table 1). A range of organic acids with different chain lengths and metal binding tendencies were also studied, including oxalate, acetate, succinate, and citrate (Table 2). Cr speciation was monitored throughout the ligand-promoted dissolution experiments to ensure that the effects of redox-independent processes were specifically isolated.

**Table 1:** Type and structures of siderophores used in this study

**Table 2:** Structures of the organic acids used in this study

### Synthesis of Cr(III)-(oxy)hydroxide

Cr(OH)_3_ was synthesized following a previous procedure^65^. Briefly, Cr(NO_3_)_3_·9H_2_O (ACS grade) was dissolved in deionized water (18 MΩ cm) to obtain a total Cr concentration of 0.1 M. The solution was slowly titrated to pH 7 with 1 M NaOH. Precipitates formed from the suspension were aged for 24 h followed by dialysis to remove remaining electrolytes. The resulting wet pastes were recovered through centrifugation, freeze dried, and finely ground. Portions of the powders were dissolved in nitric acid for Cr concentration analysis by inductively coupled plasma-mass spectroscopy (ICP-MS; Agilent 7500a). The specific surface area of the synthesized solid was determined to be 95 m^2^ g^−1^ by Brunauer–Emmett–Teller (BET) gas adsorption analysis using an Autosorb-1-MP surface pore analyzer (Quantachrome Corp.).

### Ligand solubilization experiments

The rate and extent of Cr(OH)_3_ solubilization were investigated in the presence of a single type of siderophore or organic acid, or a combination of siderophore and organic acid. The representative siderophores included DFOB (mesylate salt, Sigma-Aldrich), enterobactin (from *Escherichia coli*, Sigma-Aldrich), and pyoverdine (from *Pseudomonas putida* ATCC 12633, EMC Microcollections, Germany). The representative organic acids included acetate, oxalate, succinate, and citrate (all ACS grade from Sigma-Aldrich).

For each ligand solubilization experiment, the Cr(OH)_3_ solids were suspended in a 0.1 M NaCl electrolyte solution with 10 mM HEPES buffer (high purity grade, Sigma-Aldrich) at pH 7 and sonicated for 15 min to disperse the particles. HEPES buffer was previously shown to not influence the rates of siderophore-promoted dissolution of metal hydroxides (see, e.g., refs.^27,65,66^). For the treatments containing a siderophore, the siderophore was added to the suspensions to achieve a final concentration of 0.1 mM. For the treatments containing an organic acid, the organic acid was added to the suspension to achieve a final concentration of 1 mM. For the treatments containing both a siderophore and an organic acid, the organic acid was added 1 min prior to the siderophore addition in order to account for its potential effect on metal hydroxide dissolution during the initial exposure^67^. Treatments containing organic acids were conducted in triplicate, and treatments containing siderophores were conducted in duplicate. Due to the limitations imposed by the quantities of purchased siderophores, the volumes of each replicate for the siderophores were 50, 5, and 2.4 ml for DFOB, enterobactin, and pyoverdine, respectively. All organic acid treatments were conducted at 50 ml volume. All experiments were conducted at room temperature in the dark using amber bottles, and were constantly agitated on an orbital shaker. A reaction time of ~ 1 month was chosen based on previous studies on Cr isotope exchange rates between aqueous Cr(VI) and solid Cr(III) oxyhydroxide at pH 7^19^ and between various Cr–Cl species at low pH^7^, both suggesting significant to complete isotope exchange may occur within a few months.

Throughout the experiments, 100 μl of filtered (0.2 μm, cellulose acetete) aliquots of each sample were collected at certain time points and analyzed for total dissolved Cr concentration by ICP-MS. Cr(VI) concentrations in the filtrates were analyzed using the diphenylcarbazide assay^68^ on an UV–vis spectrophotometer (Cary 60, Agilent) and were below the detection limit (0.2 μM) for all treatments. Thus, the total dissolved Cr concentration in the filtrate was considered to be Cr(III). The pH values of the reaction suspensions stayed at 7.0 ± 0.1 throughout the entire experiment for all treatments. No microbial growth was observed for the duration of the experiment. Cr release rates were determined by fitting a regression line through the linear portion of the dissolution profile for each condition, and were normalized to BET surface area for consistency of comparison with previous studies on ligand-promoted dissolution of Cr(OH)_3_^27^.

### Cr isotope analysis

At various time points along the dissolution profile, an entire sample bottle was sacrificed for Cr isotope analysis, thus reflecting the cumulative isotope signature at that time point. The ^50^Cr-^54^Cr double spike method was used to correct for fractionation during sample purification and isotope measurement^15,69^. The double spike solution with known ^50^Cr/^54^Cr ratio (~ 1) was added as early as possible in the sample preparation process and was added in the same valence(s) as the Cr in the sample (i.e., Cr(III)). Proper amount of spike was added to the filtered samples to ensure an optimum spike/sample ratio of ^54^Cr_spike_/^52^Cr_sample_ ~ 0.5. Cr was purified from matrix ions using a cation exchange resin method modified from Bonnand et al.^70^. Briefly, the sample–spike mixtures were gently evaporated to dryness and refluxed with 6 N HCl to allow full isotopic equilibrium between spike and sample. Samples were then dissolved in 0.5 N HCl and loaded onto Bio-Rad AG50W-X8 (100–200 mesh) cation exchange resin. Cr was eluted from the resin after loading and collected for isotope analysis. Purified Cr samples were dissolved in 0.7 N HNO_3_ and measured for Cr isotopic compositions on a NeptunePlus multicollector ICP-MS. Ti, V, and Fe interferences were monitored and corrected, with corrections typically < 0.1‰. A concentration matched unprocessed SRM 979 standard was analyzed before and after every sample to monitor instrument drift. Sample values were normalized to the average values of the bracketing standards. Based on standards and duplicate preparation and analysis of various types of real samples, our analytical precision was about ±0.08‰ (95% confidence level) using 200–1000 ng Cr. The starting Cr(OH)_3_ solid was dissolved by 0.7 N HNO_3_ and analyzed for Cr isotopic composition as described above and was −0.08 ± 0.04‰. The total procedural blank ranged from 0.2 to 1 ng, which is negligible compared to the sample Cr amount; therefore, blank correction was not performed.

### Data availability

Data sets generated during and/or analyzed during the current study are available from the corresponding author on reasonable request.

## Electronic supplementary material

Supplementary Information
