# Supplementary material for: Redox-independent chromium isotope fractionation induced by ligand-promoted dissolution
Source: Nat Commun. 2017 Nov 17;8:1590. doi: 10.1038/s41467-017-01694-y (PMC5693864; doi:10.1038/s41467-017-01694-y)
Supplement: Supplementary file 1 — Supplementary Information [file 41467_2017_1694_MOESM1_ESM.pdf]

**Supplementary Table 1. Cr release rate using different normalization strategies.** Cr release rates as determined from the slope of the linear region of the dissolution profiles for each treatment (~5 days). The uncertainty was determined based on the standard deviation between replicates. Different Normalization schemes were used for comparison to previous studies on siderophore-promoted dissolution of $Cr(OH)_3$ (refs[1,2,3]) or as ligand concentration normalized rates.

| Ligand | Cr-ligand release rate | | |
|---|---|---|---|
| | $\mu mol_{Cr} \cdot d^{-1}$ | $mol_{Cr} \cdot m^{-2} s^{-1}$ | $mol_{Cr} \cdot mol_L^{-1} m^{-2} s^{-1}$ |
| acetate | 0.03±0.01 | 1.94 ±0.81 ×10$^{-14}$ | 3.88±1.61 ×10$^{-11}$ |
| acetate + DFOB | 0.04±0.00 | 2.15±0.29 ×10$^{-14}$ | 4.73±0.63 ×10$^{-10}$ |
| succinate | 0.04±0.02 | 2.73±1.1 ×10$^{-14}$ | 5.46±2.34 ×10$^{-11}$ |
| succinate + DFOB | 0.04±0.02 | 2.53±1.0 ×10$^{-14}$ | 5.61±2.27 ×10$^{-10}$ |
| oxalate | 0.11±0.01 | 6.53±0.56 ×10$^{-14}$ | 1.74±0.75 ×10$^{-10}$ |
| oxalate + DFOB | 0.14±0.03 | 7.77±1.3 ×10$^{-14}$ | 1.87±0.36 ×10$^{-9}$ |
| citrate | 0.27±0.01 | 1.67±0.04 ×10$^{-13}$ | 3.34±0.09 ×10$^{-10}$ |
| citrate +DFOB | 0.23±0.08 | 1.63±0.09 ×10$^{-13}$ | 3.59±0.20 ×10$^{-9}$ |
| DFOB | 0.08±0.00 | 4.85±0.10 ×10$^{-14}$ | 9.70±0.21 ×10$^{-10}$ |
| enterobactin | 1.27±0.07 | 7.73±0.40 ×10$^{-13}$ | 1.55±0.08 ×10$^{-7}$ |
| pyoverdine | 0.94±0.15 | 5.73±0.93 ×10$^{-13}$ | 2.29±0.37 ×10$^{-7}$ |

$$Rate\ (\mu mol \cdot d^{-1}) = \frac{[Cr]_{dissolved}\ (\mu mol{\cdot}L^{-1})}{time\ (d)}$$

$$Rate\ (mol \cdot m^{-2} s^{-1}) = \frac{[Cr]_{dissolved}\ (\mu mol{\cdot}L^{-1})}{time\ (s) \times [Cr(OH)_3]\ (g{\cdot}L^{-1}) \times Cr(OH)_3\ surface\ area\ (m^2{\cdot}g^{-1})}$$

$$Rate\ (mol_{Cr} \cdot mol_L m^{-2} s^{-1}) = \frac{[Cr]_{dissolved} (\mu mol{\cdot}L^{-1})}{time\ (s) \times [Cr(OH)_3]\ (g{\cdot}L^{-1}) \times Cr(OH)_3\ surface\ area\ (m^2{\cdot}g^{-1}) \times [Ligand]\ (mol{\cdot}L^{-1}) \times volume\ (L)}$$

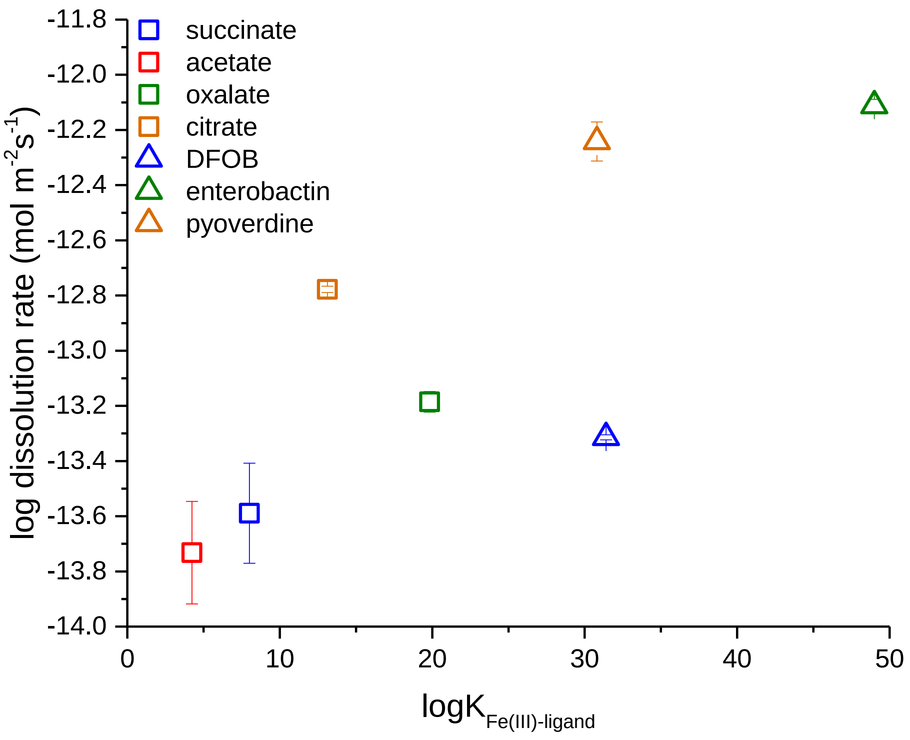


**Supplementary Figure 1. Cr dissolution rate normalized to surface area.** Initial dissolution rates of 0.2 g/L of $Cr(OH)_3$ (mol $m^{-2}$ $s^{-1}$) at pH 7 and ionic strength of 0.1 M as a function of the stability constant of the corresponding Fe(III)-ligand complexes. Stability constants for Fe(III)-organic acid and Fe(III)-siderophore complexes are the same as in Figure 4.

## Supplementary References


1. Duckworth, O. W., Akafia, M. M., Andrews, M. Y. & Bargar, J. R. Siderophore-promoted dissolution of chromium from hydroxide minerals. *Environ. Sci.-Process Impacts* **16**, 1348-1359, doi:10.1039/c3em00717k (2014).

2. Saad, E. M. *et al.* Siderophore and organic acid promoted dissolution and transformation of Cr(III)-Fe(III)-(oxy)hydroxides. *Environ. Sci. Technol.*, doi:10.1021/acs.est.6b05408 (2017).

3. Carbonaro, R. F., Gray, B. N., Whitehead, C. F. & Stone, A. T. Carboxylate-containing chelating agent interactions with amorphous chromium hydroxide: Adsorption and dissolution. *Geochim. Cosmochim. Acta* **72**, 3241-3257, doi:10.1016/j.gca.2008.04.010 (2008).
